# Eastern Europe's alcohol-related liver disease crisis is preventable

**DOI:** 10.1016/j.lanepe.2026.101742

**Published:** 2026-06-02

**Authors:** The Lancet Regional Health – Europe

The WHO European region has the world's highest alcohol consumption and one of the highest burdens of alcohol-attributable liver disease globally. Cirrhosis and liver cancer together cause nearly 780 deaths per day across the region, and liver disease has become a major source of premature mortality and working years lost. Within Europe, this burden falls unevenly, with Eastern Europe showing the highest rates of alcohol-related liver disease (ALD) and alcohol-attributable mortality. Alcohol accounts for nearly one in five premature adult deaths among men in parts of Eastern Europe, and Global Burden of Disease 2023 estimates suggest that alcohol-related harm remains disproportionately high across the region.

ALD develops silently over years, often remaining undetected until the advanced stages, but this burden is neither inevitable nor irreversible. Across Eastern Europe, ALD burden is shaped heavily by policies on pricing, availability, advertising, enforcement, and cross-border trade. Evidence from Eastern Europe shows that sustained alcohol-control policies can reduce alcohol-related harm, while weaker policies can quickly worsen outcomes.

Lithuania shows what alcohol-control policy can achieve. Its comprehensive reforms between 2016 and 2018, including higher excise taxes, reduced sales hours, stricter advertising restrictions, and an increase in the legal purchase age from 18 to 20 years, produced one of the steepest documented declines in per capita alcohol consumption and alcohol-related mortality in the Baltic region. Life expectancy increased from 71 to 77 years between 2007 and 2023, cirrhosis mortality fell by 29%, and adolescent intoxication dropped by 49% compared with five similar EU jurisdictions.

Elsewhere in Eastern Europe, however, alcohol-related harm continues to rise where sustained alcohol-control policies are absent. In Romania, alcohol consumption increased by 30% between 2013 and 2023, with a pronounced rise after the COVID-19 pandemic. Meanwhile, Estonia's reduction in alcohol excise taxes by 25% in 2019, partly in response to cross-border purchasing from Latvia, shows how national reforms can be weakened by regional market dynamics.

Across Eastern Europe, alcohol-control reforms continue to be undermined by industry interference, fragmented governance, weak enforcement, and policy inertia as highlighted in *The Lancet Regional Health–Europe* Series on Chronic Liver Disease in Europe. Even well-designed reforms can be tempered by affordability, informal markets, weak enforcement, and cross-border purchasing, especially in areas where public health institutions do not have the authority to withstand commercial and fiscal pressures. The Alcohol Preparedness Index, which assesses the strength of national alcohol-related policies shows persistently low scores and slow policy progress across parts of Eastern Europe, mirroring the region's disproportionate alcohol-attributable burden.

WHO best buys already provide the framework for reducing alcohol-related harm, from higher prices and marketing restrictions to drink-driving countermeasures and access to brief interventions and treatment of liver disease. Sustained implementation of these measures could substantially reduce the burden of ALD across Eastern Europe.

Clinical systems also require reform. Across Eastern Europe, late diagnosis and fragmented care limit opportunities for early intervention. Services for liver disease, addiction, and mental health remain poorly connected and screening programmes often have unclear routes into treatment. Earlier detection will improve outcomes only if health systems can provide coordinated multidisciplinary care.

Eastern Europe's liver disease crisis is preventable but requires political commitment equal to the scale of the burden. Governments need to consistently implement evidence-based alcohol policies, strengthen health systems to detect and treat ALD earlier, and build regulatory institutions resilient to commercial pressure. These measures should be treated not as optional public health tools, but as core components of national non-communicable disease strategies. Lithuania shows that comprehensive alcohol-control policies can change the trajectory of ALD for the better. Romania demonstrates how the continued absence of these policies can escalate the burden. ALD is one of the most preventable major causes of premature mortality in Eastern Europe; what is needed now is consistent implementation and sustained political commitment. The question is whether governments across the region are willing to act.

